# Occurrence of gustducin-immunoreactive cells in von Ebner’s glands of guinea pigs

**DOI:** 10.1007/s00418-013-1094-9

**Published:** 2013-04-19

**Authors:** Yuki Ibira, Hiroyuki Yokosuka, Maiko Haga-Tsujimura, Sumio Yoshie

**Affiliations:** Department of Histology, The Nippon Dental University School of Life Dentistry at Niigata, 1-8 Hamaura-cho, Chuo-ku, Niigata, 951-8580 Japan

**Keywords:** Guinea pig, von Ebner’s glands, Taste bud, Gustducin-immunoreactive cell, Immunohistochemistry

## Abstract

An immunohistochemical examination of guinea-pig taste buds in vallate papillae revealed gustducin-immunoreactive cells in the area of von Ebner’s glands, minor salivary glands. Since there have been no reports describing those cells in these locations for other species, we investigated these glands in order both to localize the cells and compare their immunoreactive characteristics with corresponding cells in the vallate taste buds. The gustducin-immunoreactive cells coincided with cells containing no secretory granules in the end portion of the glands, which was supported by the electron-microscopic immunocytochemistry. Double immunofluorescence microscopy confirmed these cells to be entirely immunopositive to type III inositol 1,4,5-triphosphate receptor (IP_3_R-3), phospholipase Cβ2 (PLCβ2), and villin and also partly immunopositive to neuron-specific enolase (NSE) and calbindin D-28K. The gustducin-immunoreactive cells in the vallate taste buds exhibited completely the same immunoreactivities for these five molecules. Accordingly, the present results give credence to a consideration that the gustducin-immunnoreactive cells in both locations are identical in function(s) e.g., chemo-reception.

## Introduction

Over recent decades, gustatory receptors and signal-transducing molecules have been progressively elucidated. Among molecules corresponding to the taste reception–transduction system, gustducin, a G-protein α subunit, was primarily cloned in murine taste buds by McLaughlin et al. (1992). This subunit was then revealed to be expressed in other mammalian taste buds including humans (Takami et al. 1994; Wong et al. 1996; Boughter et al. 1997; Cho et al. 1998; Ming et al. 1998; Tabata et al. 2003; Ohkubo et al. 2007).

During the course of our immunohistochemical examination of taste buds in vallate papillae of guinea pigs, gustducin-immunoreactive cells were encountered in the area of von Ebner’s glands. These glands are minor salivary glands shallowly located in the tongue radix and open their ducts at the grooved bottoms of the vallate and foliate papillae, where many taste buds exist in the epithelial layer facing the grooves. A distinct feature of the glands is their serous or sero-mucous secretions like those produced by the parotid gland, a major salivary gland (Young and van Lennep 1978). Cells immunoreactive for gustducin have already been reported outside the taste bud (Höfer et al. 1996; Höfer and Drenckhahn 1998; Merigo et al. 2005; Tizzano et al. 2006, 2010; Hass et al. 2007; Ogura et al. 2010; Krasteva et al. 2011, 2012). So far as we are aware, however, there are no articles describing such cells in von Ebner’s glands.

Our previous immunohistochemical studies on guinea pig-vallate taste buds revealed that there appeared immunoreactive cells for neuron-specific enolase (NSE), calbindin D-28K (former name: spot 35 protein), or type III inositol 1,4,5-triphosphate receptor (IP_3_R-3), as well as gustducin (Yoshie et al. 1988, 1989, 1991; Ohkubo et al. 2007). Supporting our findings, immunohistochemical studies on mammalian taste buds have clarified that gustducin-immunoreactive cells concomitantly express these molecules (reviews: e.g., Medler 2008, 2010; Ishimura 2009; Niki et al. 2010; Kinnamon 2012). Furthermore it has been elucidated that gustducin-immunopositive cells existing outside taste buds show immunoreactivity for villin (Höfer and Drenckhahn 1998) or phospholipase Cβ2 (PLCβ2) (Merigo et al. 2005; Hass et al. 2007; Mace et al. 2007; Gulbransen et al. 2008).

Since lingual taste buds and von Ebner’s glands differentiate from the epithelial cells of tongue primordium, we speculated certain qualitative relationship between the cells in the two locations. The present study therefore aimed to clarify the localization of the gustducin-immunoreactive cells in von Ebner’s glands and compare their immunoreactivities for the five molecules (NSE, calbindin D-28K, IP_3_R-3, villin, and PLCβ2) with those cells in the vallate taste buds.

## Materials and methods

Male and female guinea pigs weighing 300–400 g were used in this study. The animal treatment followed the instructions of the Institutional Animal Care and Use Committee at our university (permission No. 45).

### Tissue preparation

The animals were deeply anesthetized by an intraperitoneal injection of sodium pentobarbital (50 mg/kg body weight) and perfused through the arterial trunk with phosphate-buffered saline (pH 7.3) and successively with 4 % paraformaldehyde in a 0.07 M phosphate buffer (pH 7.3). The tissues containing von Ebner’s glands and/or vallate papillae were excised from the tongues and further immersed in the same fixative for 2–12 h at 4 °C.

For immunofluorescence histochemistry and pre-embedding electron-microscopic immunocytochemistry, the fixed specimens were rinsed overnight in the same phosphate buffer containing 30 % sucrose at 4 °C and then rapidly frozen in isopentane precooled to −35 °C. Frozen sections, 5–10 μm in thickness, were made on a cryostat, mounted on APS-coated glass slides, and processed for the following immunostaining procedures.

For the semithin sectioning of von Ebner’s glands, the fixed specimens were minced into small tissue blocks, rinsed in the phosphate buffer, dehydrated through a graded ethanol series and propylene oxide, and embedded in Epon 812. Consecutive semithin sections were cut at a thickness of 0.5 μm and mounted on glass slides by heating at 80 °C. For immunostaining, the resin was removed from the tissue sections with sodium methoxide according to Mayor et al. (1961) immediately before the immunostaining procedures.

In order to reduce the non-specific immunoreaction, each tissue section was treated with 10 % normal goat serum for 30 min at room temperature prior to the following immunostaining.

### Antibodies and antigens

This study employs six kinds of primary antibodies: two rabbit polyclonal antibodies against gustducin and PLCβ2, and four mouse monoclonal antibodies against IP_3_R-3, NSE, calbindin D-28K, and villin. The antibodies against gustducin, PLCβ2, and NSE were purchased from Santa Cruz Biotechnology (USA); the antibodies against IP_3_R-3, calbindin D-28K, and villin were from BD Biosciences (USA), Sigma (USA), and Research Diagnostic (USA), respectively. As secondary antibodies fluorescent-dye conjugated IgGs (Alexa Fluor 555-labeled goat anti-mouse IgG and Alexa Fluor 488-labeled goat anti-rabbit IgG) were purchased from Molecular Probes (USA). An avidin–biotin-complex staining kit (Histofine SAB-PO) was obtained from Nichirei (Japan).

All corresponding antigens were purchased from the primary antibody sources.

### Immunocytochemistry for gustducin in semithin sections

To detect the immunoreactivity in von Ebner’s glands, the avidin–biotin peroxidase complex (ABC) technique was adopted. Immediately after removal of Epon 812, the semithin sections were incubated with the antibody against gustducin (dilutions: 1:100–1:800) for 24 h at 4 °C, then with biotin-conjugated goat anti-rabbit IgG for 30 min at room temperature, and finally with avdin–biotin-peroxidase complex for 30 min at room temperature. The immunoreaction was visualized with 0.0125 % 3,3′-diaminobenzidine tetra-hydrochloride (DAB) and 0.004 % hydrogen peroxide in 0.05 M TRIS/HCl buffer (pH 7.6).

Neighboring semithin sections were stained with toluidine blue without removing the resin.

### Immunocytochemistry for gustducin by electron microscopy

The detection of gustducin-immunoreactive cells in von Ebner’s glands was carried out using frozen sections by the pre-embedding method. To visualize the immunoreactive cells under the electron microscope, the treated sections, having been adequately stained by the ABC method mentioned above, were successively post-fixed with 1 % osmium tetroxide in a 0.07 M phosphate buffer (pH 7.3) for 30 min. They were then dehydrated through a graded ethanol series and embedded in Epon 812. Ultrathin sections were made, stained briefly with uranyl acetate, and examined with a JEM 1200EX transmission electron microscope at an accelerating voltage of 80 kV.

### Double immunostaining for gustducin and another molecule

Using frozen tissue sections containing von Ebner’s glands and/or taste buds, light-microscopic immunohistochemistry was performed by either an indirect or direct fluorescence method.

Individual sections were incubated overnight at 4 °C with a mixture of the antibody against gustducin (1:200 dilution) and one of the antibodies against IP_3_R-3 (1:800), NSE (1:200), calbindin D-28K (1:200), or villin (1:100). After incubation, these sections were exposed to a mixture of Alexa Fluor 488-labeled anti-rabbit IgG (1:100) and Alexa Fluor 555-labeled anti-mouse IgG (1:100) for 2 h at room temperature. Since both anti-gustducin and anti-PLCβ2 polyclonal antibodies were prepared from rabbits, we used a Zeon^®^ Rabbit IgG Labeling kit (Molecular Probes, USA) for double immunostaining. After blocking non-specific binding sites with 10 % normal goat serum for 30 min at room temperature, the sections were incubated for 1–2 h at room temperature with diluted complexes of the rabbit IgGs and fluorescent dye-labeled Fab fragments (Alexa Fluor 488-labeled anti-gustducin rabbit IgG, 1:180 dilution; Alexa Fluor 594-labeled anti-PLCβ2 rabbit IgG, 1:180 dilution) which had been prepared beforehand according to the manufacture’s instructions.

These stained sections were observed and photographed under a confocal laser scanning microscope (LSM510; Zeiss, Germany) or fluorescence/light microscope equipped with a digital imaging system (Axioplan/AxioCam; Zeiss, Germany).

### Specificity controls of the immunoreaction

All immunoreaction specificity was checked using the following three negative controls: omission of the primary antibodies, incubation of tissue sections with normal host animal sera in place of the primary antibodies, and incubation of sections with the primary antibodies which had been preincubated at 4 °C for 24 h with the corresponding antigens at 10–100 μg/ml.

## Results

**Fig. 1:**
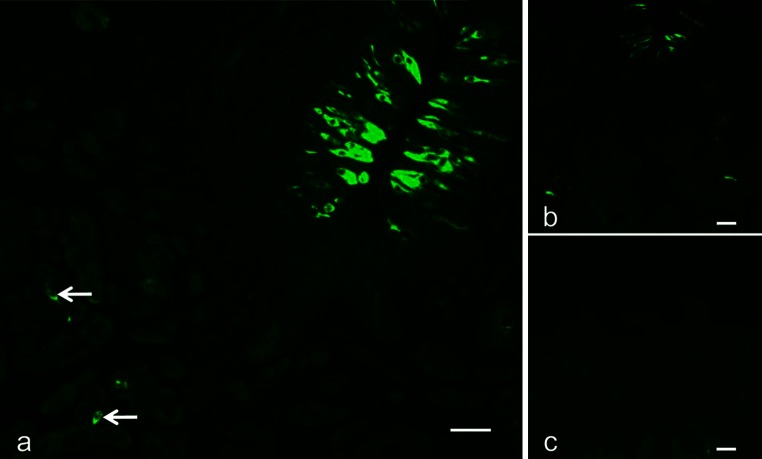
**a**–**c** Fluorescence-microscopic immunohistochemistry for gustducin in the tongue radix including vallate papillae and von Ebner’s glands. **a** Immunoreactive cells are discernible not only in the vallate taste buds (*upper right*) but also in the deeper part of the glands (*arrows*). **b**, **c** Consecutive sections immunostainined with the non-absorbed primary antibody (**b**) or with the antibody preabsorbed with the corresponding antigen (**c**). The Fluorescence vanishes from the tissue section immunostained with the preabsorbed antibody (**c**). *Scale bars* 50 μm

All the antibodies used in this study exhibited positive immunoreactivity in parts of cells in both the vallate taste buds and von Ebner’s glands. When the tongue sections containing the vallate papillae and von Ebner’s glands were immunostained for gustducin, immunoreactive cells were also found in von Ebner’s glands (Fig. 1a).

The immunohistochemical reactions after the three control procedures mentioned above were all negative, thus confirming the specificities of all immunohistochemical findings (Fig. 1b, c).

### Localization of gustducin-immunoreactive cells in von Ebner’s glands

**Fig. 2:**
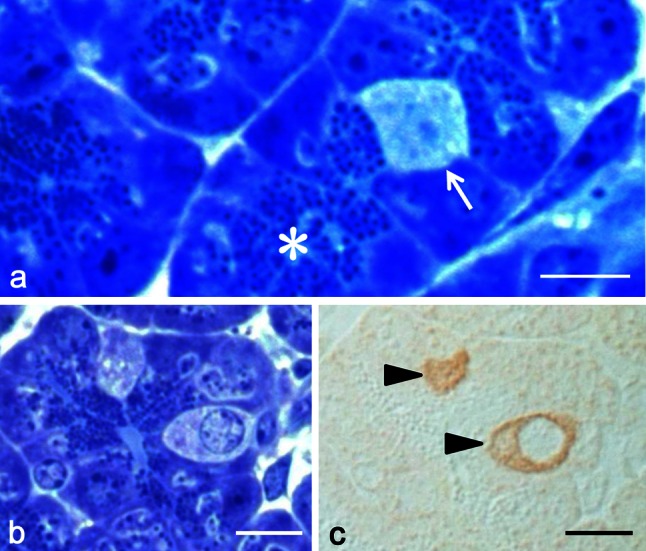
**a**–**c** Semithin sections of von Ebner’s glands stained with toluidine blue (**a**, **b**) or immunostained for gustducin (**c**). **a** End portion of the glands. Predominant are the secretory cells accumulating secretory granules in the supranuclear cytoplasm (*asterisks*). A clear cell intermingles with the secretory cells (*arrow*). Note that the clear cell lacks secretory granules. **b**, **c** Consecutive sections. The clear cells exclusively exhibit the immunoreactivity for gustducin (*arrowheads*). *Scale bars* 10 μm

**Fig. 3:**
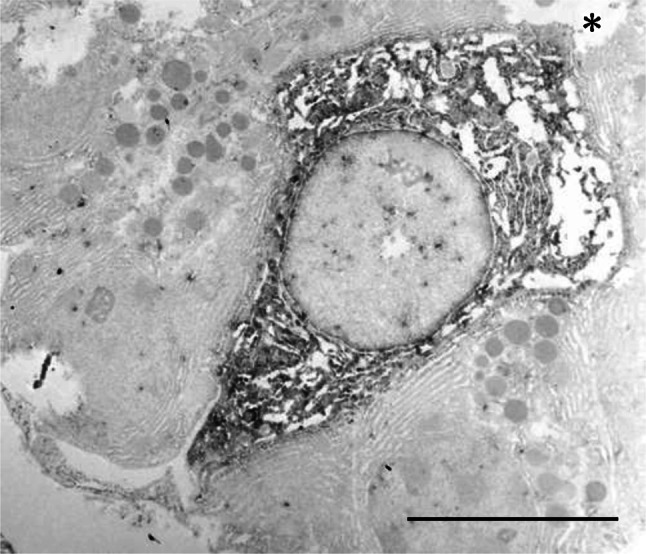
Electron-microscopic immunocytochemistry for gustducin in von Ebner’s glands. The immunoreactivity is localized in the whole cytoplasm of a cell lacking secretory granules, which is a significant feature of the clear cell. The cell extends from the basement membrane to the apical lumen (*asterisk*). *Scale bar* 6 μm

Observation of 0.5-μm semithin sections of von Ebner’s glands stained with toluidine blue readily confirmed that the end portion, tubulo-alveolar in shape, consisted of typical serous cells accumulating densely stained secretory granules in the supranuclear cytoplasm (Fig. 2a). Careful examinations often revealed cells with different aspects in the end portion. These cells appeared clear, being oval to triangular in profile, and contained no secretory granules (Fig. 2a, b). They usually existed solitarily in the end portion. All the clear cells exhibited immunoreactivity for gustducin (Fig. 2b, c). In support of these light-microscopic findings, the electron-microscopic immunocytochemistry showed that cells immunopositive to gustducin did not contain any secretory granules in the supranuclear cytoplasm (Fig. 3). Although we have not yet examined precisely the fine structure of the glands by conventional electron microscopy, the immunoreactive cell developed endoplasmic reticulum-like structures in the whole cytoplasm and contained a round, large, and electron-lucent nucleus (Fig. 3). These structural features resembled those of the Type II cell in the vallate taste bud of guinea pigs (Yoshie et al. 1990). However, we failed to detect the gustducin-immunoreactive cells forming microvilli at the apical surface.

In summary, the gustducin-immunoreactive cells in the glands were solitarily located in the end portion and intermingled with secretory cells.

### Immunohistochemical characteristics of the gustducin-immonoreactive cells

We applied immunohistochemistry for the molecules of NSE, calbindin D-28K, IP_3_R-3, PLCβ2, and villin to von Ebner’s glands and the taste buds for comparing the immunoreactivities of the gustducin-immunoreactive cells between the two locations.

**Fig. 4:**
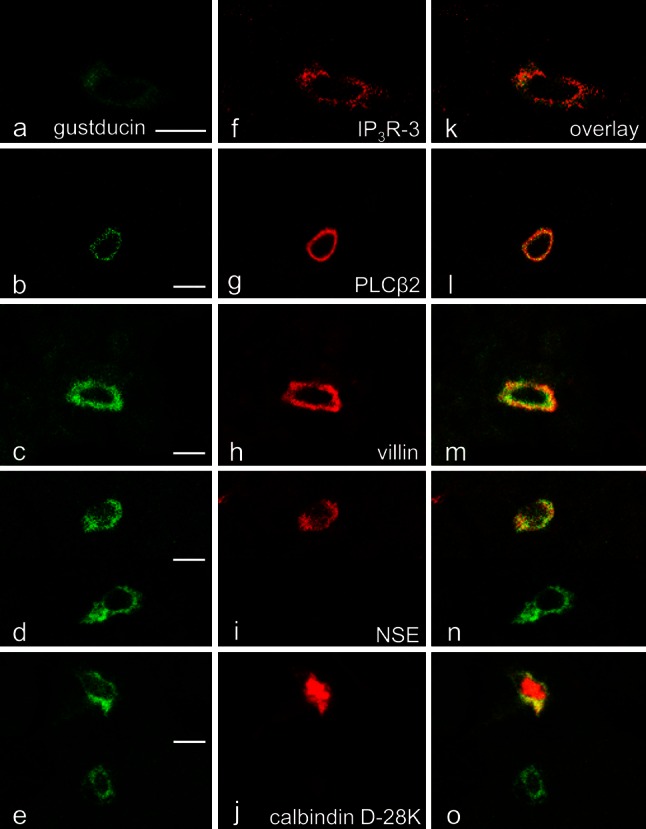
**a**–**o** Laser-scanning microscopic immunohistochemistry for gustducin (**a**–**e**) and the others (**f**–**j**); overlays of **a**–**e** and **f**–**j** (**k**–**o**) in von Ebner’s glands, respectively. The gustducin-immunoreactive cells are all immunopositive to IP_3_R-3 (**k**), PLCβ2 (**l**), and villin (**m**) but only partly immunopositive to NSE (**n**) and calbindin D-28K (**o**). *Scale bars* 10 μm

The gustducin-immunoreactive cells in von Ebner’s glands were entirely immunopositive to IP_3_R-3, PLCβ2, and villin; they are partly immonopositive to NSE (69 % of the cells) and calbindin D-28K (72 %) (Fig. 4). Moreover, all the cells immunopositive to IP_3_R-3, PLCβ2, or villin were concomitantly immunoreactive for gustducin. These immunohistochemical data are summarized in Table 1.

**Table 1:** Immunoreactivities of gustducin-immunoreactive cells in both von Ebner’s glands and vallate taste buds

Immunoreactive for				
IP_3_R-3	PLCβ2	Villin	NSE	Calbindin D-28K
+	+	+	+/0	+/0
(68)	(50)	(37)	(66/30)	(48/19)

Numbers in parentheses indicate total counts of cells appearing in von Ebner’s glands, which was obtained through the observation of 98-tissue sections from eight animals

+ reactive, 0 non-reactive

**Fig. 5:**
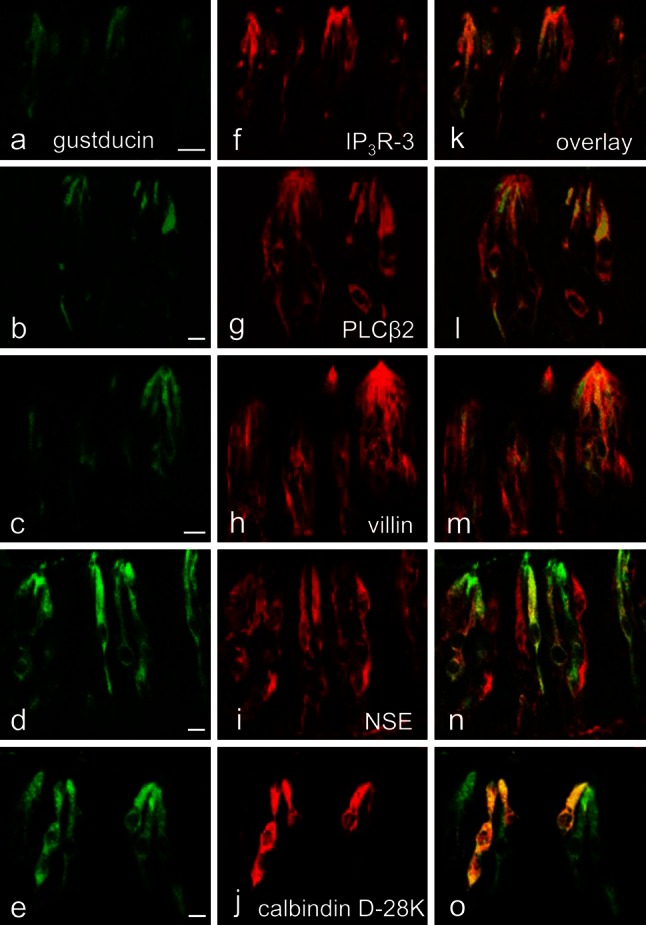
**a**–**o** Laser-scanning microscopic immunohistochemistry for gustducin (**a**–**e**) and the others (**f**–**j**); overlays of **a**–**e** and **f**–**j** (**k**–**o**) in vallate taste buds, respectively. The gustducin-immunoreactive cells are all immunopositive to IP_3_R-3 (**k**), PLCβ2 (**l**), and villin (**m**) but only partly immunopositive to NSE (**n**) and calbindin D-28K (**o**). *Scale bars* 10 μm

In the vallate taste bud, those cells likewise were entirely immunopositive to IP_3_R-3, PLCβ2, and villin and partly immunopositive to NSE and calbindin D-28K (Fig. 5; see also Ohkubo et al. 2007). Being viewed reversely, all the IP_3_R-3-, PLCβ2-, or villin-immunopositive cells were not always immunoreactive for gustducin, which was different from the immunohistochemical data obtained in von Ebner’s glands (Fig. 5).

## Discussion

This study has demonstrated the existence of gustducin-immunoreactive cells as a constituent of the end portion of von Ebner’s glands in guinea pigs. Light and electron microscopic investigations have been performed in these glands for several mammalian species, except for the guinea pig (Hand 1970; Takeda 1976; Toyoshima and Tandler 1986; Azzali et al. 1989; Gargiulo et al. 1995; Cheng et al. 2009; Isola et al. 2010; Yahiro et al. 2011). However, such non-granulated cells have not been described in the literature.

In contrast, cells exhibiting immunoreactivity for gustducin have been detected in certain regions other than the taste buds. These cells were described as being distributed in the digestive system (Höfer et al. 1996; Höfer and Drenckhahn 1998; Hass et al. 2007) and the respiratory system (Merigo et al. 2005; Tizzano et al. 2006). In the former organs, the gustducin-immonoreactive cells appear in the epithelia of the duodenum and pancreatic duct (Höfer et al. 1996; Höfer and Drenckhahn 1998), as well as in the stomach (Hass et al. 2007). Moreover, Höfer et al. (1996) were first to reveal that the gustducin-immunoreactive cells are identical with the cells named brush cells. Gustducin was further demonstrated to be expressed in the identical cells of the pancreatic duct (Höfer and Drenckhahn 1998).

The brush cell, initially described in the rat tracheal mucosa by Rhodin and Dalhamn (1956), apically forms a characteristical tuft of microvillous processes. A conspicuous ultrastructural feature of the tracheal brush cell is its accumulation of membrane-bound vesicles or granules at the basal cytoplasm just like the basal granulated endocrine cells in the intestine, strongly suggesting the chemosensory and paracrine or endocrine functions of the brush cell (Taira and Shibasaki 1978).

In their immunohistochemical study on the rat digestive and respiratory systems, Höfer and Drenckhahn (1992) were first to reveal that the brush cells express villin and fimbrin, which are bridging molecules of the cytoskeletal protein, F-actin. In addition to the brush cell, villin is expressed also in Merkel cells (Toyoshima et al. 1998) and taste bud cells (Yoshie et al. 2003).

The present study could not clarify whether the gustducin-immunoreactive cells in von Ebner’s glands possess microvilli at their apices. Since it is well known that chemo-sensory cells such as gustatory cells and brush cells form microvilli, and express villin, the present gustducin-immunoreactive cells are expected to possess microvilli. We are now pursing this question under the conventional transmission electron microscope.

T1Rs and T2Rs, taste receptors originally cloned in taste buds, have been demonstrated to be expressed in the gustducin-immunoreactive cells locating outside the taste organ (Wu et al. 2002; Mace et al. 2007; Margolskee et al. 2007; Gulbransen et al. 2008; Kaji et al. 2009). Accordingly, functional evidence of their being *taste*-sensing chemosensory cells is accumulating for the corresponding cells (reviews: Rozengurt and Sternini 2007; Finger and Kinnamon 2011; Kinnamon 2012).

Because of their topographical proximity, functional relationships between the taste buds and von Ebner’s glands have long been argued. These glands produce serous or sero-mucous secretions and open their ducts at the grooved bottoms of vallate and foliate papillae (Young and van Lennep 1978). Consequently, it is generally accepted that the secretions of the glands wash and refresh the taste-bud apicies, or taste hairs, where tastant receptors are localized.

More recent studies have suggested much closer relationship between the taste buds and von Ebner’s glands (review: Sbarbati et al. 1999). The authors propose another relationship between the both as follows: the vallate papilla–von Ebner’s gland complex, which may indicate the involvement of the taste buds in the secretory control of the glands. Actually, considering the ultrastructural features of canine taste buds in the vallate papilla, Kanazawa (1993) hypothesized that transmitters may be released from the base of the Type III or gustatory cell and possibly exerts paracrine and endocrine effects, suggesting von Ebner’s glands as a target. Such a relationship was speculated between the brush cells and the pancreas or liver (Höfer and Drenckhahn 1996, 1998; Sbarbati et al. 1999).

The present study revealed that the immunoreactivities of the gustducin-immunopositive cells in von Ebner’s glands completely coincided with those of the corresponding cells in the taste buds as follows: all the cells concomitantly immunopositive to IP_3_R-3, PLCβ2, or villin; parts of the cells concomitantly immunopositive to NSE or calbindin D-28K. Focusing on gustducin, IP_3_R-3, NSE, and calbindin D-28K, our previous studies of the guinea-pig vallate taste bud strongly suggested that the Type III cell is immunoreactive for these four molecules and the Type II cell for the three molecules excluding calbindin D-28K (Yoshie et al. 1988, 1989, 1991; Ohkubo et al. 2007). From the present results, therefore, it could be reasonable to consider that the gustducin-immunoreactive cells in both locations are identical in function(s). Actually ultrastructure of the gustducin-immunoreactive cell in von Ebner’s glands more or less resembled that of the Type II cell in vallate taste buds (Yoshie et al. 1991).

Although we have not yet detected taste receptors in the gustducin-immunoreactive cells of either location, their target candidates are the neighboring secretory cells and autonomic nerves by the paracrine mode, and the sensory nerves by forming synapses. An ultrastructural examination of these glands will be essential to disclose the exact nature or function of the gustducin-immunoreactive cells.
